# Immunoepigenetic Regulation of Inflammatory Bowel Disease: Current Insights into Novel Epigenetic Modulations of the Systemic Immune Response

**DOI:** 10.3390/genes14030554

**Published:** 2023-02-23

**Authors:** Guillermo Bastida, Alejandro Mínguez, Pilar Nos, Inés Moret-Tatay

**Affiliations:** 1 Inflammatory Bowel Disease Research Group, Health Research Institute La Fe (IIS La Fe), 46026 Valencia, Spain; 2 Gastroenterology Department, Inflammatory Bowel Disease Unit, La Fe University and Polytechnic Hospital, 46026 Valencia, Spain

**Keywords:** Immunoepigenetic, IBD, Crohn’s disease, ulcerative colitis, PBMC

## Abstract

The immune system and environmental factors are involved in various diseases, such as inflammatory bowel disease (IBD), through their effect on genetics, which modulates immune cells. IBD encompasses two main phenotypes, Crohn’s disease, and ulcerative colitis, which are manifested as chronic and systemic relapse-remitting gastrointestinal tract disorders with rising global incidence and prevalence. The pathophysiology of IBD is complex and not fully understood. Epigenetic research has resulted in valuable information for unraveling the etiology of this immune-mediated disease. Thus, the main objective of the present review is to summarize the current findings on the role of epigenetic mechanisms in IBD to shed light on their potential clinical relevance. This review focuses on the latest evidence regarding peripheral blood mononuclear cells and epigenetic changes in histone modification, DNA methylation, and telomere shortening in IBD. The various identified epigenetic DNA profiles with clinical value in IBD could be used as biomarkers for more accurately predicting disease development, treatment response, and therapy-related adverse events. Ultimately, the information presented here could be of potential relevance for future clinical practice in developing more efficient and precise medicine to improve the quality of life for patients with IBD.

## 1. Introduction

Inflammation is an endogenous response to pathogens and injuries, producing beneficial physiological responses when the inflammation causes minimal tissue damage but prevents triggering harmful effects [1,2]. However, inflammation can manifest as a dysregulated and pathological response that can progress to a chronic form, resulting in the onset and perpetuation of various disorders [2], including inflammatory bowel disease (IBD).

IBD is a chronic relapsing gastrointestinal inflammatory condition with a continuously increasing incidence [3,4], which includes two major diseases: Crohn’s disease (CD) and ulcerative colitis (UC). They share analogous characteristics, such as intermittent, chronic, and/or progressive inflammation and features specific to each such as transmural inflammation of the gastrointestinal wall (from the mouth to the anus) in CD and inflammation limited to the colon and rectal mucosa in UC [5]. Although the etiopathogenesis of IBD remains unknown, the disease appears to be a dysregulated immune response to unknown triggers in genetically predisposed patients [6].

In recent decades, studies have focused on the reductionist paradigm of a single altered component in IBD (mostly centered on an altered gene) as responsible for dysregulated pathological inflammation [7]. Genetic analyses of patients with CD and UC have long shown familiar clustering, although in the absence of a clear Mendelian inheritance pattern [5]. This finding shed light, in the beginning, on the understanding of IBD pathogenesis and helped identify molecular mechanisms involved in IBD, which assisted in developing effective anti-inflammatory therapies [7]. In this regard, data have suggested the contribution of genetics to IBD, from monogenic to allogenic and polygenic. The very early-onset IBD (onset in the first several years of life) had been related to a more monogenic defect [8]. However, most cases of IBD have shown no clear familial association and have been considered more polygenic [8]. Interestingly, the reported risk ratios for siblings of patients with CD and UC, when compared with the general population, have shown percentage ratios between 15% and 42% for CD and between 7% and 17% for UC [5], indicating that the two entities have slight differences in genetic susceptibility, being more pronounced in CD.

### 1.1. Genetic Analysis

Omics employ technological advances such as next-generation sequencing, high-throughput data generation, and molecular networks to assess changes in a biological system [7]. The use of omics can help in understanding the etiopathogenesis of diseases, mainly complex diseases such as IBD [9]. Concerning this point, genome-wide association studies, along with meta-analyses by international IBD consortia, have identified more than 200 IBD susceptibility loci [10]. Furthermore, approximately 70% of the genetic loci associated with IBD are shared by a range of immune-mediated inflammatory diseases (IMIDs), which supports the concomitance of IBD and other autoimmune disorders [8]. Specific genetic loci related to T-cell differentiation and inflammation have been found, such as interleukin (IL)-23/T-helper (Th) 17 signaling, host-microbe interactions, autophagy, and integrin activation, which are associated with IBD [2,7].

Large-scale deep resequencing and whole genome sequencing studies are revealing new rare low-frequency variants and functions of genome-wide study loci in IBD [7,11]. By analyzing samples from 30,000 patients, a study concluded that the binary classification of IBD can be further delineated to include three distinct entities: ileal CD, colonic CD, and UC [12]. Although many of these polymorphisms are located in noncoding regions of the genome, transcriptional regulatory functions have also been attributed [12,13,14]. This is an unsurprising finding, given that the coding sequence of genes represents approximately only 2% of the whole genome, and the remaining 98% is noncoding DNA, with significant but yet unknown potential functions [5]. Considering also their role in important cellular pathways, these IBD-related genetic variants explain only a small proportion of disease risk [7].

One major challenge remains to be unraveled, namely, the fact that only a proportion of patients with IBD respond to treatment, and this response and the disease’s clinical progression can differ among patients. It is, therefore, clear that the etiology and heterogeneity of IBD are more complex and multifactorial than initially believed [2,4,7]. Moreover, elements outside of the protein-gene axis demonstrate their role in the pathogenesis of IBD [5]. In the posthuman-genome era, the focus is on identifying the factors that influence genetic variations as an adaptation process to various environmental triggers [7,11,15]. Thus, environmental factors and their relationship to the genome-epigenome are considered primary causes of IBD [7]. In this context, precision medicine has become an important issue in clinical practice [16], in which the definition of the role of epigenetics in IBD can provide new directions toward achieving better therapies for and monitoring IBD [17,18].

### 1.2. Epigenetics

Epigenetics can be defined as inheritable molecular events (in a reversible and cell-type-specific manner) that can regulate DNA-related processes. Epigenetics includes chemical modifications to DNA (methylation and demethylation) without changing the underlying DNA sequence; modifications to histone proteins, which are components of the nucleosomes that DNA wraps around; and the action of noncoding RNAs such as microRNAs [12]. Epigenetics is therefore considered a regulator of the complex machinery behind inflammatory disorders, contributing to the expression of inflammation-associated genes [10] and is therefore associated with IBD pathogenesis [7,19,20].

One of the factors considered important in modulating the immune system is the self-regulated intestinal microbiota [8], which is influenced by the environment and can, in turn, influence intestinal epithelial cells and resident and migrated immune cells. Due to its complexity, it is an interesting factor that needs to be explored in detail in other specific reviews. On the other hand, noncoding RNAs, especially microRNAs, represent the most studied epigenetic mechanism in IBD, and many reviews have dealt with this topic [6,19]. Therefore, we have focused on describing in detail other more novel and less studied epigenetic mechanisms in IBD.

**Figure 1:**
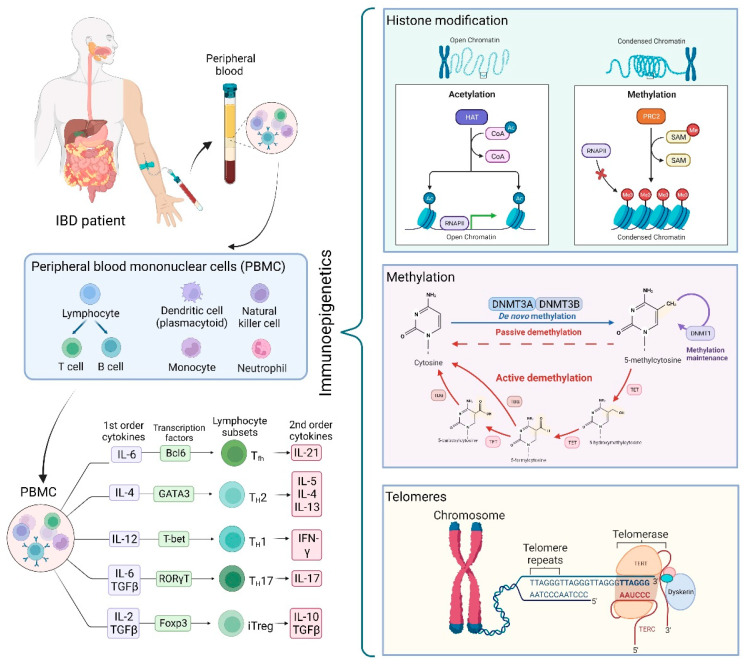
Summary of the mechanisms detailed in the review of the biological context and epigenetic processes of methylation, histone modification, and telomere dysfunction.

Understanding how epigenetic modifications modulate the inflammatory processes in IBD and their contribution to disease progression and to the response to various therapeutic approaches could help in better managing IBD [21]. This review covers the current evidence regarding the effects of methylation, histone modification, and telomere dysfunction in the systemic immune response in IBD (Figure 1).

## 2. Histone Modifications and Chromatin Organizers as Influencers of the Impaired Immune System in IBD

In eukaryotic cells, DNA is wrapped around a histone octamer, forming nucleosomes, the basic unit of chromatin [22]. Epigenetic modifications regulate (directly or indirectly) chromatin compaction and accessibility to transcription factors [23]. When histone is loosely attached to DNA, the chromatin is called euchromatin, which favors DNA transcription. When histones are compressed, they form heterochromatin, which prevents DNA transcription [24].

Modifications to the histone tails are mediated by various types of enzymes, which can be classified as epigenetic writers (add a mark), readers (interpret the mark), and erasers (remove the mark) [22]. Other types of histone modifications include ubiquitination, phosphorylation, glycosylation, and citrullination, although acetylation and methylation are the most studied [24].

The acetylation of lysine (K) residues on histone proteins by histone acetyltransferases (HATs, which are writers) results in the suppression of positive charges, thereby making DNA more accessible to transcription factors (euchromatin). Histone deacetylases (HDACs, which are erasers) induce strong links between DNA and histone proteins (heterochromatin), preventing the binding of transcription factors [23]. In IBD, HDAC inhibitors increase acetylation levels, resulting in the reduction of colitis in murine models, with a decline in proinflammatory cytokines and the migration of inflammatory cells to the intestinal site [5].

Histone methylation is coordinated by lysine methyltransferases (KMTs) using S-adenosylmethionine as a methyl group donor, which can result in monomethylation, dimethylation or trimethylation in lysines, monomethylation or dimethylation in arginine residues [22]. Histone methylation produces a more site-specific effect, i.e., the methylation mark can result in either a positive or negative effect on transcription depending on where and how much methylation is produced in the histone tails [23]. For example, the di/trimethylation of lysine 4 of histone 3 (H3K4me2/3) favors transcription, whereas di/trimethylation of lysine 27 of histone 3 (H3K27me2/3) represses transcription [23]. Unlike acetylation, methylation functions as a docking site for recruiting other factors (e.g., in bromodomain PHD finger transcription factor, BPTF) [22].

### 2.1. Histone Modification and Immune Mediators of Inflammation in IBD

In IBD, the current evidence highlights the dysfunction of toll-like receptor (TLR)-mediated innate immunity as a central player in the pathogenesis of IBD [15]. TLR signaling is marked by phosphorylation of histone 3 at serine 10 (H3S10), methylation at H3K4, and acetylation at H3K9/K14, which results in the induction of inflammation-related response genes, such as IL-6, IL-8, MCP-1, and IL-12p40 [2]. These elements are responsible for the importance of the innate immune response in the induction of gut inflammation [25,26].

**Figure 2:**
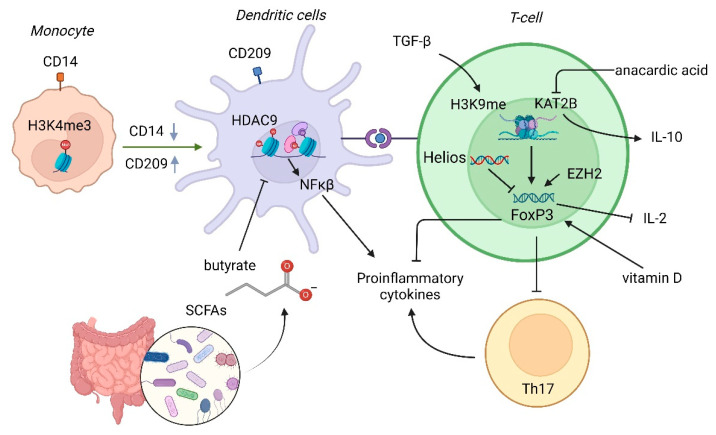
Histone modifications as influencers of the impaired immune system in IBD. H3K4me3: trimethylated lysine 4 of histone 3; HDAC: histone deacetylases; SCFA: short-chain fatty acid; H3K9me: methylated lysine 9 of histone 3; KAT2B: lysine acetyltransferase 2B; EZH2: histone methyltransferase enhancer of Zeste homolog 2.

Butyrate, the short-chain fatty acid (SCFAs) produced by the intestinal microbiota’s fermentation of undigestible fibers, functions as an HDAC inhibitor by HDAC9 inhibition, which enhances histone H3 acetylation in the *NOD2* promoter region [5]. Butyrate has immunomodulatory functions (Figure 2), regulating intestinal inflammation by cell surface G-protein coupled receptors, but its levels are reduced in IBD [26,27]. In addition, the first gene identified to confer susceptibility to IBD was *NOD2/CARD15*, especially in terms of susceptibility to CD [28]. It is, therefore, plausible that regulating this gene through a pharmacological approach of butyrate supplementation might reduce inflammation (reduced nuclear factor kappa β signaling) and improve the integrity of the intestinal epithelium [5]. SCFAs can also modulate histone acetylation to regulate the conversion of immunoglobulin-type B cells in health and disease [24], which could be of importance in IBD.

Crohn’s disease has been associated with the deregulated production of interferon (IFN)-γ, IL-6, and IL-12, while ulcerative colitis has been associated with IL-5 and IL-13 [29]. In both diseases, IL-10 has been classically considered an anti-inflammatory cytokine, preventing gut inflammation, with the ability to suppress innate and adaptive inflammatory responses (by reducing the numbers of Th1, Th17, natural killer, and macrophage cells) and boost regulatory responses [30]. IL-10 can, in turn, be secreted by various immune cells (e.g., CD4+ T cells and macrophages) and is regulated by lysine acetyltransferase 2B (KAT2B) [10], which is downregulated in the inflamed colonic mucosa of patients with IBD [10,31]. Anacardic acid (a major component of cashew nut shells) plays a role in inhibiting KAT2B, with a reduction in H4 lysine 5 (H4K5) acetylation levels in the *IL-10* promoter [32]. This process reduces IL-10 expression [5], which can disrupt the innate and adaptive inflammatory responses (Figure 2) [31]. Other cytokines, such as transforming growth factor (TGF)-β, can potentially promote Th1 conversion to proinflammatory Th17 [10] and even induce regulatory T-cell development [33]. TGF-β modulates the histone lysine methyltransferase G9A, which in turn methylates H3K9 at the promoters of *IL-17α*, *IL-17f*, *Rorc*, and *Foxp3* [10]. The loss of G9A increases response to TGF-β1 and, therefore, Th17 and regulatory T-cell (Treg) differentiation [10,34]. Through SMAD4 and histone deacetylation regulation, TGF-β attenuates its characteristics (epigenetic, transcriptional, and functional characteristics) to prevent chronic intestinal inflammation [33].

Among the transcription factors, Helios (a member of the Ikaros transcription factor family) stands out because of its role in T-cell functions through the recruitment of histone modifiers (Figure 2). In murine models, Helios depletion causes reduced HDAC recruitment with increased *IL-2* promoter activity and reduced forkhead box P3 (Foxp3) [35]. This process should be taken into account, given that HDAC inhibitors can prevent colitis by boosting Foxp3+ levels [36]. In this context, the HDAC inhibitor suberoylanilide hydroxamic acid, which is known as vorinostat and has been approved for cutaneous T-cell lymphoma treatment [37], can reduce the production of the proinflammatory cytokines IL-6 and TNF-α, which protects against colonic inflammation in a murine model [38]. Suberoylanilide hydroxamic acid can also inhibit the mobilization and accumulation of inflammatory cells in the gut [37].

Chromatin-organizing complexes, such as CTCF (CCCTC-binding factor) and condensins, facilitate the generation of DNA loops in which DNA regulatory elements are positioned close to gene promoters, which can modify gene expression [10]. T-cell activation in response to IL-2 and T-cell receptor engagement induces changes in the chromatin structure in the condensing complex (CAP-H2); however, changes in this complex produce T-cell growth impairment and preserve the quiescent state [10]. In line with this, the effect of excessive IL-2 signaling has been related to a predisposition to very early-onset colitis [39]. NCAPD2 and three subunits of condensin have a primary role in microbial immunity and its recognition by human intestinal epithelial cells, although their impaired expression can lead to proinflammatory cytokine production [40].

Another factor to consider, due to its important epigenetic influence and relation to IBD, is vitamin D (Figure 2), which has a role as an immune modulator by increasing FoxP3 and IL-10 and suppressing the inflammatory cytokines IL-17, IFN-γ, IL-21, and IL-22 [28,41,41]. Low blood vitamin D levels have been linked to the risk of relapse and exacerbation in IBD [41,42]. Vitamin D can be regulated by histone modification, which has been considered one of the most important epigenetic mechanisms. In fact, the monoubiquitination of histone H2B results in inflammation through the decrease in vitamin D receptor activity [24].

### 2.2. Histone Modification and Immune Cells in IBD

The immune system comprises innate and adaptive immune cells. The former (e.g., macrophages, dendritic cells, neutrophils, natural killer cells) produce cytokines and chemokines that initiate inflammation and activate the adaptive immune system [43], which in turn (T and B cells, mainly) are highly specific and confer long-lasting immunity [25]. Within the adaptive immune cells, Th1 cells produce large amounts of IFN-γ, whereas Th2 cells release IL-4, IL-5, and IL-13. However, the development of Th1 and Th2 is epigenetically regulated by the chromatin modification of the IFN-γ gene [44]. An abnormal Th1 immune response has classically been considered responsible for intestinal inflammation in Crohn’s disease, whereas UC was considered more a Th2-mediated disease. However, this paradigm has changed, and other novel lymphocyte subpopulations (Th17, Th9, Th22, Tr1, and Tfh cells) are demonstrating their role in IBD [45]. Thus, a shift from the classical Th1/Th2 to the novel Th17/Treg has been proposed [29]. Th17 is an important source of IL-21, an IL-2-related cytokine that is upregulated in IBD [25]. *IL-21* expression can be epigenetically repressed by reducing chromatin accessibility, and this blockade can, in turn, downregulate IL-17 and IFN-γ production in IBD [46].

Among the CD4+ T cells, the Th1, Th2, and Th17 effector cell subsets are important defenders against pathogens, whereas CD4+ Tregs help control the activity and proliferation of effector cells. In the context of IBD, effector CD4+ T-cell hyperactivity and Treg development defects have been continuously observed [10]. Concerning this point is the histone methyltransferase enhancer of Zeste homolog 2 (EZH2), which helps maintain a regulatory phenotype in lymphocytes. However, EZH2-deficient FoxP3+ T cells secrete proinflammatory cytokines and lead to spontaneous IBD in murine models [47]. EZH2 has shown decreased expression in patients with IBD (Figure 2) [5], and the loss of EZH2-FoxP3 interaction in Tregs is thought to compromise Treg physiology and intestinal inflammation, which can remain chronically activated [48]. EZH2 is also a critical epigenetic determinant in preventing colitis through the modulation of the TNFα-dependent inflammatory response by H3K27me3 [49].

The Jumonji domain-containing protein 3 (JMJD3), a histone demethylase, regulates T-cell differentiation by transcription repression. In murine models, the loss of JMJD3 promotes differentiation of Th2 and Th17 while inhibiting Treg and Th1 differentiation, thereby contributing to reduced overall inflammation [10]. JMJD3 has therefore been considered a potential epigenetic target for treating IBD [50,51]. Interestingly, the epigenetic initiation of Th17 is also linked to methylation on the *IL-6* receptor promotor (H3K4me2/3), resulting in the activation of the *IL-6/STAT3* signaling pathway, which in turn inhibits Treg development but augments Th17 cell stability and maturation [23].

The differential methylation of the speckled 140 kDa protein (SP140) observed in the peripheral blood mononuclear cells of patients with CD, as well as the fact that reduced *SP140* expression in intestinal biopsies correlates with a good anti-TNF response, suggests a role for SP140 in IBD [52]. SP140 functions as an epigenetic reader, the inhibition of which can prevent the generation of inflammatory macrophages as well as the regulation of proinflammatory and CD-related gene expression [52,53]. Homologous to the speckled-protein family, the autoimmune regulator AIRE is a master regulator of central tolerance, which links transcription factors and epigenetic machinery by driving the expression of tissue-specific T-cell antigens. The AIRE loss-of-function can give rise to autoimmune diseases, with a potential role in IBD [54].

In addition to adaptative immunity, the epigenetic influence on innate immunity has also been observed. Specifically, monocytes, which can differentiate into dendritic cells through *CD14* and *CD209* genes, have epigenetic control of differentiation with an impact on their cellular functions [55]. Monocytes and dendritic cells are regulated by the epigenetic modification of the histones H3K4me3 and H3K9ac at the *CD14* promoter and H3K4me3 and H3K9ac at the promoter and body of the *CD209* gene [55], which have major repercussions on IBD pathogenesis [56].

## 3. Methylation as a Mechanism for Imprinting Altered Key Elements in IBD

DNA methylation can regulate DNA expression by controlling the accessibility of the transcription machinery to the binding sites and chromatin state [7]. By the covalent addition of a methyl group to the DNA cytosine, catalyzed by DNA methyltransferase (DNMT) in a cytosine-guanine dinucleotide (CpG), gene expression is often repressed. Hypomethylation could lead to the activation of dormant repeat elements followed by the aberrant expression of associated genes (Figure 1) [1]. The various identified DNMTs can help maintain the pre-existing DNA methylation profiles or participate in de novo DNA methylation [24]. However, many DNA methylation changes have occurred within introns and intergenic sequences for which their functional significance remains unidentified [7]. DNA methylation represents the most stable, easy-to-use, and studied (along with microRNAs) epigenetic mechanism [24,57].

### Impaired Methylation Results in the Alteration of Immune Factors

Impaired DNA methylation has become the hallmark of most diseases, including IBD and cancer, and there is an essential need to discover the methylation signature involved in these diseases [1]. Over the past decade, a strong correlation has been established between DNA methylation and IBD pathogenesis. Differentially methylated positions, regions, and genes have been associated with various IBD phenotypes in peripheral blood leukocytes [58]. A recent study established epigenetic evidence of a north-south gradient in IBD [59], observing replication of former epigenetic results for *VMP1* (vacuole membrane protein, also known as *TMEM49*, which plays a regulatory role in autophagy) and *SBNO2* (strawberry notch homolog 2, which has a cellular response to IL-6 and macrophage activation) in a new cohort of Scandinavian and UK patients, but not in a Spanish cohort. However, the epigenetic results from *RPS6KA2* (ribosomal protein S6 kinase A2, which has a regulatory role in cell growth and differentiation) were replicated in all three cohorts [59]. These results show that the differences in methylation due to the exposome (i.e., the location of a specific population) are an issue that remains to be explored.

DNA methylation regulates cytokine expression during the T-cell differentiation process. In the IBD context, genes involved in the downstream signaling of IL-23, such as *STAT3*, oncostatin-M (*OSM*), and *STAT5*, present impaired methylation profiles in CD [60], which could explain why these immune system mediators are altered in IBD. *IFN-γ* and *IL-4* genes also present a differential methylation profile during Th1 and Th2 differentiation, respectively, in normal conditions and during an IBD flare-up [24]. Focusing on CD4+ memory T cells, in healthy subjects, RAR-related orphan receptor-C (*RORC*) genes and genes encoding ligands for P-selectin and E-selectin are hypomethylated, in contrast to the hypermethylation in naïve CD4+ T cells [61]. In IBD serum, however, levels of those adhesion molecules are low at remission, but E-selectin levels are higher in active CD. The potential role of methylation in the adhesion elements of the immune system and disease activity has been reported [62].

In normal conditions, CD8+ memory T cells show low methylation levels of *IFN-γ* and *IL-2* genes [60]. In IBD (either during a flare-up or during inactivity), however, CD8+ lymphocytes have an increased proliferative response to IFN-γ stimuli [63]. In addition, defects in the gene encoding of the methyl-CpG-binding domain protein 2 (*MBD2*) result in differentiation defects in CD8+ T cells, thereby influencing gene expression and resulting in impaired effector and memory CD8+ T cells [64]. This process alters the inflammatory capacity of murine CD11c+, which results in increased colitis severity [65]. The novel identification of disease-associated T cell clonotypes in the CD8+ T cell population in IBD [60,66] highlights the importance of studying these cells in intestinal disorders.

Hypermethylation of TNF receptor-associated factor 6 (*TRAF6*) in peripheral blood mononuclear cells from patients with IBD decreases its expression. *TRAF6* is a gene that mediates signal transduction downstream from the TNF receptor superfamily and the Toll/IL-1 family [24,67]. In experimental models, the lack of *TRAF6* expression was related to exacerbated colitis [68]. Other hypermethylated genes in active CD include homeobox protein engrailed-1 (*EN1*), Wilms tumor protein (*WT1*), and fibroblast growth factor receptor 2 (FGFR2), whereas neurogenic locus notch homolog protein 4 (*NOTCH4*) was hypomethylated with respect to CD in remission [60]. Regarding other implicated elements, GSK-J4 (a selective inhibitor of the histone demethylase JMJD3/UTX) attenuated inflammatory colitis by reducing the inflammatory potential and increasing the tolerogenic features of CD [51].

Recently, an epigenetic signature with clinical value for the noninvasive diagnosis of CD has been reported [57]. In our study, the differential methylation profiles of the antimicrobial peptide α-defensin 5 (*DEFA5*) and *TNF* genes were recorded in patients with CD at onset and during inactivity, demonstrating that this signature is maintained regardless of disease activity and is therefore related to the disease’s chronic nature. The methylation status of *DEFA5* (gain of methylation) and *TNF* (loss of methylation) genes could be useful as biomarkers to characterize patients with CD.

Another element to consider is *TAP1* (transporter 1, an ATP-binding cassette subfamily B member), which has a role in generating a cytotoxic T-cell response through human leukocyte antigen class I proteins and presents differential methylation in CD [59]. Furthermore, a novel aspect is the study of 5-hydroxymethylcytosine, i.e., the hydroxymethylation of cytosine and its oxidized derivative, which is receiving attention as a new epigenetic factor due to its as yet unknown potential role in the development of diseases such as IBD [24].

## 4. Telomeres in the Context of the Immune System and IBD

Telomeres are specific heterochromatic structures located at the end of linear chromosomes. The main function of telomeres is to preserve genomic stability to ensure the chromosome’s integrity and complete replication [69,70]. The stability and activity of telomeres can classically be maintained through the shelterin protein complex and by alternative lengthening of telomeres [70]. These structures are especially interesting in the context of the immune system because they are implied in the dynamic cellular network, suggesting that telomere maintenance is critical (Figure 1) [71]. Thus, immune competence depends on the rapid expansion of clonal T and B cells, and therefore impaired telomeres can influence defective immune responses [70]. A deeper analysis of telomere content in the adaptative response has revealed that B cells have the highest telomerase activity, followed by CD4+ and then CD8+ T cells [72].

The factors that stand out in the modification of telomere length include epigenetic mechanisms, the presence of reactive oxygen species, and inflammatory reactions [71]. DNA methylation and histone modifications of the telomerase catalytic subunit (*TERT*) gene promoter change the expression of this gene [70], with important biological implications in disease. The relationship between IBD and telomeres was established after observing shorter telomeres in the intestinal epithelium of patients with UC. However, this was considered more a consequence of oxidative stress during the inflammatory process than a triggering factor [73]. It has been proposed that the shorter leukocyte telomeres of patients with UC might reflect oxidative damage secondary to inflammation [74]. A recent publication, however, reported that telomere dysfunction could initiate the inflammatory process in IBD through activation of the transcription coregulator YAP1 and proinflammatory cytokines such as IL-18 [73].

Classically, telomere shortening has been linked with dysplasia and neoplasia in ulcerative colitis [74,75]. A study by Truta B et al. found no clear link between telomere shortening in anticipation of IBD, although the authors stated that more studies are needed with larger sample sizes to confirm or refute their findings [75]. Nevertheless, age-related telomere loss could contribute to immune dysfunction and the autoimmune response and could therefore explain the onset of IBD after middle age. This process would affect the lymphoid more than the myeloid lineage (granulocytes and monocytes), given that, in normal conditions, these patients show low levels of telomerase activity [70].

## 5. Other Aspects to Consider

In light of the above, there are additional aspects that should be considered. First, there is growing evidence showing a relationship between differential DNA epigenetics and ethnicity. Studies should therefore be conducted with diverse populations to replicate the previous findings [1]. Second, factors such as age, sex, lifestyle, comorbidities, and pharmacological treatments can affect the epigenetic signature and should therefore be considered when designing studies, as should the careful selection of patients and controls [76], given that, e.g., loss of the methylated genome occurs with age [24]. Third, another important challenge in IBD epigenetic studies is to determine whether peripheral blood or mucosal biopsy is more representative of reality, given that the cell-type heterogeneity of these specimens can affect the results [76]. Currently, epigenetic signatures are thought to be cellular- and tissue-type-specific. Therefore, results reflecting inflammation-related DNA epigenetic changes could be influenced by a single cell type component [1]. Employing purified disease-specific cell types before analysis could prevent difficulties in interpreting the data. However, as several cell types have been linked to the pathogenesis of IBD, the selection of disease-specific cell types in IBD is a real challenge [76]. Thus, the complexity of the experimental analysis leads researchers to employ non-purified samples. In addition, the complex experimental protocols employed to obtain purified cells could bias the results and, therefore, ultimately fail to clarify the current uncertainties. Therefore, the use of peripheral blood as a sample source to identify and study the epigenetics of IBD still offers many advantages over other samples, such as intestinal tissue, mainly due to the ease of cell retrieval, storage, and handling.

## 6. Conclusions

The impact of environmental factors on the pathophysiology of IBD is gaining importance in terms of determining which epigenetic changes influence the outcome, disease progression, and response to IBD treatments. The development of new, cutting-edge research tools for epigenetic studies allows the possibility of identifying specific profiles of histone modifications, methylation signatures, and/or telomere shortening with clinical value in IBD. Furthermore, the various epigenetic mechanisms are, in many cases, interlinked. This interrelationship should be explored to better understand the influence of epigenetics on IBD pathogenesis. The discovery of biomarkers based on epigenetics could help monitor disease activity and, with the selection of therapeutic interventions, ultimately lead to advances in the care of patients with IBD.

## Data Availability

Not applicable.
